# The Sforzesco brace can replace cast in the correction of adolescent idiopathic scoliosis: A controlled prospective cohort study

**DOI:** 10.1186/1748-7161-3-15

**Published:** 2008-10-31

**Authors:** Stefano Negrini, Salvatore Atanasio, Francesco Negrini, Fabio Zaina, Gianfranco Marchini

**Affiliations:** 1 ISICO (Italian Scientific Spine Institute), Via R. Bellarmino 13/1, 20121 Milan, Italy; 2 Vita e Salute University, Via Olgettina, Milan, Italy; 3 Centro Ortopedico Lombardo, Via Passeroni 6, Milan, Italy

## Abstract

**Background:**

The conservative treatment of adolescent idiopathic scoliosis (AIS) has traditionally been divided into two phases–correction and stabilisation–and casts, even if less used today, can be considered the best standard in the correction phase. Till the present, however, no comparison between cast and brace efficacy has been proposed.

**Methods:**

This is a prospective cohort study with a retrospective control group. The aim was to verify if it is possible to obtain with a specifically developed rigid brace results comparable to a cast. We considered fifty AIS patients who had refused surgery, aged 14.1 ± 1.5 years, with 46.7 ± 7.8° Cobb scoliosis. Thirty-two consecutive patients (with no drop-outs) were prospectively followed up with the Sforzesco brace (SBG), and compared against a retrospective group of eighteen patients treated with the Risser cast (RCG). The treatment time (the total correction phase) was 19 ± 3 months. Out-of-brace x-rays were compared, as well as clinical results.

**Results:**

Compliance and hours of treatment were higher in the RCG while all the other parameters were not different. We observed a reduction of 6° Cobb and an important aesthetic gain in both groups (P<0.05). Three patients (6%) worsened, while 56% improved (36% at least 10°, and 14% 15° or more). The SBG did show results comparable to the RCG, with only minor differences in terms of scoliosis correction. On the contrary, straightening of the spine (decrease of the sagittal physiological curves) was much higher in the RCG but was not clinically significant in the SBG.

**Conclusion:**

In the corrective phase of AIS treatment it is possible with a specific rigid brace (Sforzesco – SPoRT concept) to obtain scoliosis correction similar to cast. Due to the human and social costs of casting, and worst sagittal profile results, Sforzesco brace should be the preferred method wherever possible.

## Background

**Figure 1:**
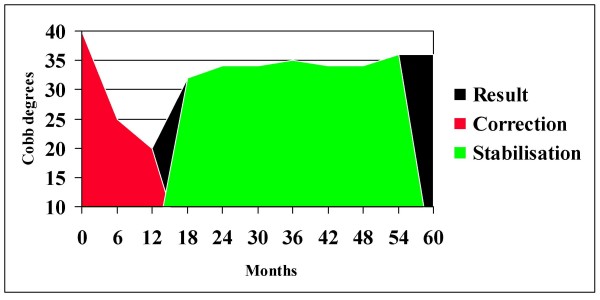
**Phases of scoliosis correction**. Correction of scoliosis according to the traditional theories [1-5]. The first stage is the corrective phase, which is followed by the stabilisation phase. In this successful case the reported values correspond to in-cast x-rays (six months and twelve months) and then out-brace ones (from eighteen to sixty months). The last result corresponds to the final x-ray at the end of treatment, when follow-up starts.

The treatment of adolescent idiopathic scoliosis (AIS) has traditionally been divided into two phases, correction and stabilisation (Figure 1), in which the correction phase has been considered to be performed through the use of casts [1-5] and stabilisation through the use of bracing. Today casts are used less than in the past, even if there are some recent papers published in the literature [6-13] and they are still used for AIS correction in a number of main Scoliosis Centres in Europe: we are personally aware of three Centres only in Milan, and at least ten other in Italy, while we have been informed of six to ten in France, two or three in Spain, one in Israel and in Poland. In other countries, like in US, England and Japan, casts are used only for Juvenile Idiopathic or Secondary Scoliosis. In many places they have been abandoned, not because of proofs of inefficacy (there aren't), but for other possible causes like: they were used for most difficult cases nowadays considered surgical; the preparation of casts is complex, time-consuming and costly (in-patient treatment, hand-made by physicians); it has a high impact on the patient's quality of life [14]; the possible side effects are important, from cast syndrome to skin problems; unbelief on the efficacy of bracing. Nevertheless, even today when bracing is criticized by many [15,16], there is a general belief that, if orthosis could be worthwhile, casts should be more effective than plastic braces in the corrective phase [7,9,11]. Due to these reasons, cast can be considered a possible high standard reference for the correction phase of AIS treatment and serve as a control group for any brace of high efficacy eventually proposed for AIS correction.

Recently a new brace has been proposed–the Sforzesco brace–whose efficacy in the short term has been shown in comparison with the Lyon brace [17,18]. This orthosis has been developed with the potential goal of substituting casts in the correction phase of AIS treatment. Nevertheless, in our perspective not using a treatment of high efficacy (cast) that we used until now [7,8,13], possibly eliminating an important instrument against high-degree AIS and increasing the rate of surgery, only because of comfort for the patient and ease for the physician, definitely required a scientific proof. Our clinical aim was to decide if abolishing Risser cast treatment was possible thank to the Sforzesco brace, or if we had to go back to casting AIS as we used to do until 2004.

The objective of this paper is to present the results obtained through our comparison of all the first patients we treated with the Sforzesco brace against those we previously had with the Risser cast in the corrective first phase of AIS treatment.

## Methods

### Design

This is a prospective cohort study with a retrospective control group. It is a best clinical practice study, because both therapies compared were considered by the treating physicians and teams (which was the same for all patients) as the best possible treatments for their patients at the time in which they had been applied. The length of follow-up for this study was the entire period of the correction phase.

### Population

We included fifty AIS patients (forty females, ten males), mean age 14.1 ± 1.5 years; the Risser sign (RS) median was 2 (distribution was RS 0: 15; RS 1: 7; RS 2: 6; RS 3: 10; RS between 3 and 4 – different on the two sides: 12); thirty-six females were post-menarche since 1.6 ± 1.1 years: in a preliminary study we verified that in Italy menarche appears very early and it's not a reliable sign of maturity, therefore we do not consider this parameter as a reason not to treat patients and we rely only on skeletal signs; average weight was 52.8 ± 10.3 kg, height 161.4 ± 7.8 cm. All patients were at their first evaluation at our institute and were at high risk of surgery (or had already refused it), and this treatment was considered the last chance in order to avoid fusion. In fact, 40% had been already treated with other orthosis but worsened. All patients with curves over 50° Cobb were first sent to surgical evaluation, and were treated only if surgery was firmly refused by the patient and his/her family, after informed consent and careful explanations of possible drawbacks of their choice (possible drawbacks of conservative treatment relate to its failure and include the prolonged use of brace without result, and a reduced surgical correction due to acquired rigidity). In all evaluations of these patients during treatment the surgical option was discussed.

The average deformity was 46.7 ± 7.8° Cobb, with an ATR (Angle of Trunk Rotation) of 12.9 ± 4.5° and corresponding hump height of 19.1 ± 6.7 mm. The Aesthetic Index [19] was 4.6 ± 1.3 on a six-point scale. The patients were divided into two groups, according to the treatments they had received. One group (SBG) included all the first thirty-two consecutive patients (twenty-five females, seven males), recruited between January 2004 and January 2006, and prospectively followed up with the Sforzesco brace; the second group (RCG) included all eighteen patients (fifteen females and three males) treated with the Risser cast we had retrieved in our database. They started their treatment between January 1998 and July 2003. Further analyses have been performed considering two sub-groups defined according to the bone age: Risser sign 2 or less (R2), versus Risser sign 3 or more (R3).

### Treatments

#### The Risser cast

**Figure 2:**
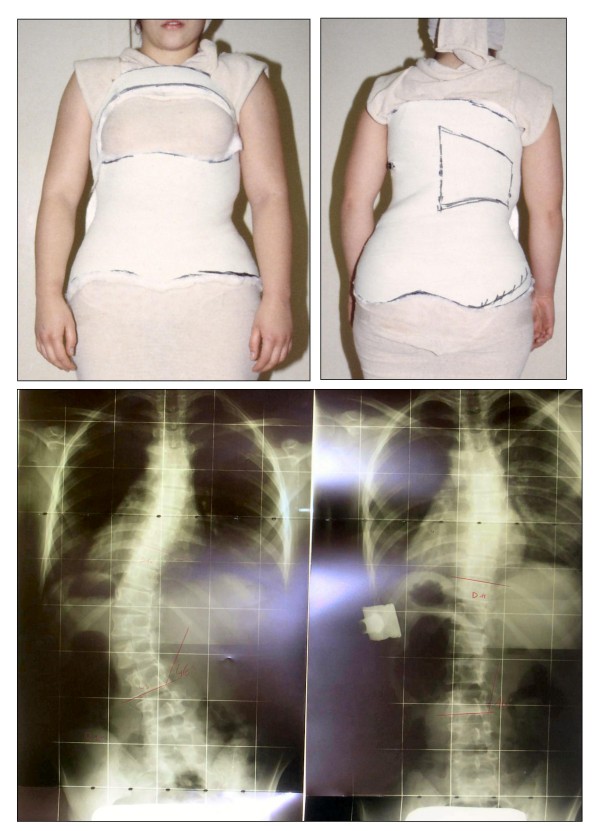
**The Risser cast**. The Risser cast for a thoraco-lumbar left curve, in the antero-posterior and postero-anterior views. An example of in-brace x-ray primary correction is reported.

The Risser cast (Figure 2) has been manufactured during an in-patient treatment according to the traditional Risser description [4,20]. We performed three casts every four months to cover one year of treatment [7,8], then followed by a period of four to six months in which the patients wore a traditional Lyon brace [2]. Finally the patients were required to perform their first x-ray without a brace in order to verify the real correction obtained in the corrective phase. The "correction phase" time in the RCG was 19 ± 3 months. The RCG has been followed-up until today, for 6.6 ± 1.8 years: the treatment time for the 11 patients who completed treatment until now was 4.6 ± 1.1 years, while the others are in treatment since 5.5 ± 1.2 years.

#### SPoRT concept: the Sforzesco Brace

**Figure 3:**
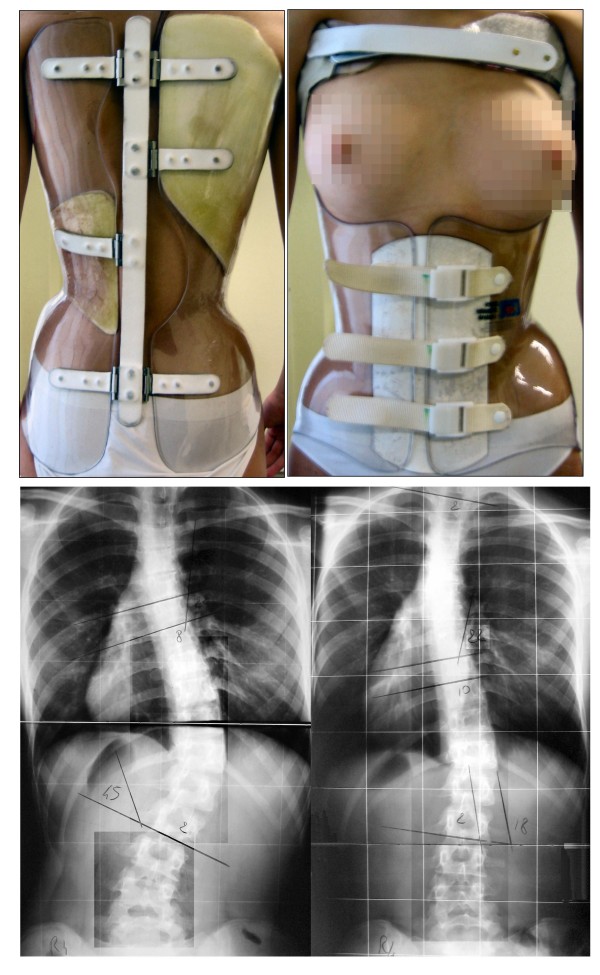
**The Sforzesco brace**. The Sforzesco brace for a thoracic right lumbar left curve, in the antero-posterior and postero-anterior views. An example of out brace x-rays results after six months is reported.

The Sforzesco brace (Figure 3) is a custom-made TLSO manufactured during outpatient treatment according to the SPoRT concept (Symmetric, Patient-oriented, Rigid, Three-dimensional, active) [17,18,21]. Particularly, in comparison with a cast, the characteristic of rigidity is important due to the material, as well as to the fact that it is shaped using only two large pieces. All patients wore the brace 23 hours per day in the first six months of treatment (twelve months for many patients), followed by progressive reduction over the next year. To compare with the RCG corrective phase, patients of the SBG were evaluated when they performed their second x-ray without brace, usually after eighteen months of treatment (the first one was performed after six months). The "correction phase" time in the SBG was 19 ± 4 months. All patients in the SBG are still in treatment since 2.7 ± 0.6 years.

#### Exercises

All patients in both group performed exercises and consequently we had two sub-groups: SEAS exercises [22,23] and usual physiotherapy. The patients decided by themselves whether they preferred to be treated according to our protocol of exercises (SEAS) or by a rehabilitation center or single physiotherapist of their choice (Usual Physiotherapy).

The main goals of SEAS (Scientific Exercises Approach to Scoliosis) in case of bracing full-time are: elimination or reduction of side effects caused by immobility (muscular hypotrophy), or the brace itself (reduction of sagittal curves, mainly kyphosis, and breathing impairment) and accentuation of brace corrective pushes [13,24,25]. Such goals are pursued through specific therapeutic modalities, subdivided into treatment phases: we describe here only those used in this study. Preparation for bracing [26]: We request the execution of exercises aimed at increasing the range of motion of the spine on all planes, so as to allow the brace to exert the maximum possible correction. We also continue proposing mobilisation exercises in the first phase of brace wearing, when it is worn for at least 21 hours per day. Brace wearing period: We initially propose exercises of "wriggling out of supports" by using the upper and lower limbs so as to facilitate adaptation to brace usage for the recommended number of hours. We require the execution of: modelling exercises in order to increase brace pressure on humps [13]; muscular endurance strengthening exercises, requiring lumbar lordosis and thoracic kyphosis preservation, while frontal and cross-sectional plane correction is guaranteed by brace pushes. We propose specific breathing activation exercises only when we detect some significant reductions of vital capacity.

The Usual Physiotherapy participants performed many different exercise protocols at a local facility according to what was preferred by their single therapist: In most cases these were in a group context, while in all cases they lasted forty-five to ninety minutes and were performed two or three times per week as in the SEAS sub-group. In some cases, the patients were required to repeat their exercises daily at home.

### Outcome criteria

The outcome criteria we considered were:

- Out-brace x-ray results measured according to Cobb degrees (a difference of 6° being considered a significant variation) [27-34];

- Clinical results measured in terms of ATR (Bunnell degrees) (a difference of 3° being considered a significant variation) [9,35-38];

- The height of the hump, whose repeatability has been proved [38], is measured in mm, and a difference of 5 mm has been considered a significant variation [39];

- In the sagittal plane, the distance from the plumbline (tangent to the apex of thoracic kyphosis) has been measured in cm at the C7 (C7P) and L3 (L3P) vertebrae; the Sagittal Index (SI) had been computed as the average of the two distances; and the intra-examiner repeatability is 0.9 cm for C7P and 1.2 cm for L3P [40]. Consequently, a difference of 1 cm for C7P, and 1.5 cm for L3P and SI, was considered a significant variation;

- The Aesthetic Index [19], which is the sum of three items (shoulders, scapulae and waist symmetry) evaluated on a three-point (0-1-2) scale to give a total of six points, was evaluated in terms of repeatability [19], and accordingly variations were considered if there was a change of at least two points.

Even if we had no measuring system for the time of brace wearing, we estimated, according to what was declared by the patients and family at each medical evaluation during a careful inquiry:

- The actual hours per day of bracing;

- The total hours of bracing during all treatment (calculated in days);

- The compliance, calculated as the percentage hours per day of bracing versus what was prescribed.

These measurements were much more accurate for RCG, where a cast had to be worn all day long consecutively without any possibility of removing it, for nearly twelve months in the first period of treatment.

All patients were evaluated always by the same treating physician. We performed reliability studies of all measurements performed: most have been previously reported [19,38,40], while Cobb degrees intra-observer reliability ranged between 2° and 4° Cobb according to the physician considered [41].

### Statistical analysis

Statistical analysis was performed, after evaluation of the distribution of variables, through ANOVA and t-test (paired for in-group comparisons, and unpaired between groups), Mann-Whitney, Fisher's Exact and chi-square tests. The statistical significance was set with α = 0.05.

## Results

We found no significant differences between the two groups at the start of treatment in all clinical and radiographic parameters nor in the total duration of treatment (19 months both groups), while, as expected, the amount of brace wearing was statistically different: 522 ± 159 versus 428 ± 126 (-10%) days in total, and 22.3 ± 2.4 versus 20.1 ± 3.7 (-18%) hours per day at the final evaluation in RCG and SBG, respectively. Moreover, the difference in compliance was significant: 99.0 ± 3.4% versus 89.3 ± 8.5 respectively. In the "corrective phase" considered we did not have any drop-out, while a compliance rate lower than 80% was reported by 4 patients, all in the SBG (12.5%).

**Figure 4:**
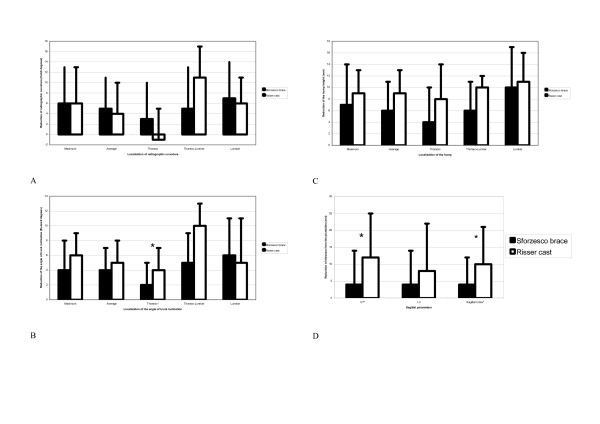
**Main results**. All clinical and radiographic parameters (a, b, c) reduced significantly in both groups with treatment, while there were no significant differences according to the orthosis used (Risser cast or Sforzesco brace) for scoliosis, and differences were excluded for thoracic ATR (b). On the contrary, the Risser cast had significantly reduced C7 distance from the plumbline and Sagittal Index (d) compared to the Sforzesco brace.

In both groups we had statistically (and clinically) significant reductions of all considered parameters with treatment (See additional file 1). Generally, in terms of Cobb degrees, for SBG the better the results the more distal the curve (Figure 4), while for RCG the outcomes have been more composite, with the best results achieved for thoraco-lumbar curves, while thoracic ones did not change statistically. Moreover, changes in the sagittal plane (they were straightening, which means worsening), even if statistically significant, were clinically relevant only for RCG (more than 1 cm flattening) but not for SBG. It is interesting to note that the thoracic reduction of ATR and rib hump were higher in RCG than in SBG.

Comparing the two groups (See additional file 2), the only differences were in the thoracic ATR, in C7P and SI, which were all reduced more in RCG than in SBG. Looking at results in single patients (See additional file 3), only two patients in SBG and one in RCG worsened (6% in total) by 7°, 8° and 10° respectively, while more than 50% improved by 6° or more, mainly in SBG. Particularly, the best results have been obtained in the most severe curve of each single patient: 36% (six in RCG and twelve in SBG) improved at least 10°, while 14% (two in RCG and five in SBG) improved at least 15°. The clinical results were better than the radiographic results, specifically in terms of aesthetics, even if the side effect of sagittal straightening of the spine was quite common, particularly in RCG (being statistically significant for SI), but also in SBG at around 50% of cases. The thoracic reduction of ATR was confirmed to be higher in RCG than SBG.

In the RCG 7 patients are still in treatment, 1 went to surgery and 1 dropped out; in the SBG we had no surgical patients yet, but 2 dropped-out.

Looking at the influence of skeletal maturity (Risser test) on results, we did not find any difference according to the bone age of the patient (See additional file 4), nor to pre-menarchial and post-menarchial status. Conversely, we verified that the patients treated with SEAS exercises statistically had significantly better clinical results than the usual physiotherapy in terms of ATR (a decrease of 6° in SEAS versus 3.5° in usual physiotherapy) and hump (-9.7 mm in SEAS versus -5.0).

## Discussion

The results of this study show the efficacy, in the correction phase, of the Risser cast and the Sforzesco brace. Only three patients (6%), out of a series of fifty with an average of 47° Cobb curves, worsened during a period of eighteen months of maximal effort of correction, while more than 50% reduced their maximal curve by at least 6° (and 14% by 15° or more). The Sforzesco brace did show results comparable to the Risser cast, having only minor differences in terms of scoliosis correction but major differences in terms of the reduction of specific spinal side effects. In fact, even if the sagittal parameters were reduced in both braces, the straightening of the spine was much higher (threefold for C7 and twofold for L3) in RCG, while it was not clinically significant in SBG.

Deepening the interpretation of our data, we must take note of the connection between three results appearing in almost all analysis:

- The reduction of thoracic ATR and rib hump is higher in RCG than in SBG;

- The flattening of the spine is higher in RCG than in SBG;

- The thoracic curve reduction is higher in SBG than in RCG.

The above data clearly suggests that the correction action of the Risser cast is too posterior while the SPoRT concept [17,18] of the Sforzesco brace is better balanced, since it serves to reduce the side effects while giving raise to better frontal-plane results with nevertheless good aesthetic ones (rib hump and Aesthetic Index).

The fact that thoraco-lumbar curves, as well as ATR and hump, have been better corrected in RCG than SBG should be carefully considered in the future, because this could suggest a possible subgroup of patients in which the mechanism of action of the Risser cast is more suitable than the SPoRT concept. Moreover, as a secondary result, in this study we confirmed what we had previously found regarding the higher efficacy of SEAS exercises versus the usual physiotherapy [26,42].

Interestingly, splitting the results according to maturation parameters did not show any difference between the sub-groups (See additional file 4). A possible explanation of this unexpected result is that our data relate only to the correction phase, and that few patients are considered. Nevertheless, we do really lack more data on this respect, and future papers should carefully look at this point.

One question could be if the results of the "correction phase" remain with time and if these patients go to surgery or not. Provisionally, we can say today that 1 out of the 18 patients in the RCG went to surgery, while 1 dropped out. These results can be explained with a selection bias: in fact many of these patients already decided not to go to surgery before starting treatment. Another explanation could be that, until now, no patient in RCG worsened, while 67% improved of 5° or more: the average result at this point of treatment is a reduction of the worst curves of 7.8 ± 7.5° Cobb, with no difference according to bone age nor to treatment finished or not; 3 patients, still in treatment, have more than 50° Cobb curves (5 at start of treatment).

In the SBG, we have until now 2 drop-outs, that in a worst-case analysis should be considered as failures, while all the other patients are still in treatment and did not went to surgery (today 5 patients are over 50° Cobb versus 9 at start of treatment). The average result at this point of treatment is a reduction of the worst curves of 7.5 ± 7.5° Cobb, with a rate of improvement of 72% and of worsening of 9%: in SBG the rate of worsening is higher in R2 group (16%). These results must be considered totally provisional because patients are still in treatment, even if we will analyze them thoroughly in future studies.

Compliance to bracing is considered a key issue today [43,44], even if measuring systems are still research tools [45-49] and not yet ready for everyday clinical usage, as should be needed in a study like this. Moreover, the RCG was a retrospective group and was therefore treated some years ago. Anyway, we must consider that in the RCG we inevitably had the highest compliance (99%) because for nearly twelve out of eighteen months of treatment the patients could not physically avoid wearing their casts, and after that the motivation is usually very high. The declared compliance obviously was not the same in the SBG (-10%), where we found all the 4 bad compliers: at any rate this was the new treatment, in which we also had fewer total hours of therapy due to the medical prescription and characteristics of a brace versus a cast. Because the compliance with the brace is really not known without some type of monitor, it is possible that the real compliance versus what was declared by the patients was much less, as has recently been shown [48], but this could have been an influence on the RCG for six months and on the SBG throughout the entire study. Nevertheless, the results for the two groups were totally comparable, and this gives even more strength to what was achieved in the SBG.

We are aware of very few studies in the indexed literature that compare the results of different concepts of bracing: These have shown the superiority of Boston over Charleston [50], TLSO over Milwaukee and Charleston [51], Chêneau over SpineCor [52], and the similarity of Milwaukee and Boston with a metal over-structure [53]. All these studies related to patients with average Cobb angles of less than 35°. We recently proved the short-term superiority of the Sforzesco brace (SPoRT concept) over the Lyon (three-point concept) in a group of patients refusing fusion, with very-high-degree scoliosis (45 ± 7° for worst individual curves, 40 ± 10° for all curves) [17]. To our knowledge, this is the first study to compare the efficacy of a brace versus a cast for AIS correction.

The main advantages of this study include the prospective data collection in the SBG, where all treated patients have been included. The fact that we have used a best clinical practice approach, because both treatments were as of that time considered the best possible ones by the treating team (an assessment that did not change during the study), along with the fact that we used a complete team approach, including treatment through [9,54]. On the other hand, limitations include: the retrospective collection of data in RCG, but this is the only way to have a best clinical approach; not having included the dropouts, but this was not possible in the retrospective RCG, so that the study could not include an intent-to-treat analysis; the absence of data on reducibility of the curves through lateral bending radiographs, avoided in patients not surgically treated; and the fact of being focused only on the corrective phase (i.e., short-term results). In this regard we must consider that research in the field of bracing cannot be limited only to final results, otherwise we will have no possibility of understanding where we might possibly fail or where we have to focus in order to increase our knowledge and ability to treat patients. Obviously these results are not the final ones, but the corrective phase of AIS treatment is the starting one; and presumably the better the results of this phase, the better the final ones [3]. The objective of this paper was not to prove the efficacy of bracing or casting but to verify whether the Sforzesco brace could be considered a valid option to substitute the Risser cast in this corrective phase of AIS treatment.

## Conclusion

Today it is possible to substitute the Risser cast in the corrective phase of AIS treatment with a specific rigid brace (Sforzesco brace), as developed according to the SPoRT concept. Future research should focus on which patient could benefit more from one instrument versus the other, because sub-grouping has provided some clues to the possible differences. Anyway, due to the human and social costs of casting, bracing should always be the preferred treatment method.

## Competing interests

The authors declare that they have no competing interests.

## Authors' contributions

All authors made substantial contributions to conception, design and acquisition of data; they have been involved in drafting and revising the manuscript; they have given final approval of the version to be published.

## Consent

Written informed consent was obtained from the patient for publication of this case report and accompanying images.

## Supplementary Material

Additional file 1 Final outcomes in the two groups. In both groups all final outcomes were significantly better than the baseline values, apart from Cobb degrees for thoracic curves for the Risser cast and thoraco-lumbar curves for the Sforzesco brace. Click here for file

Additional file 2 Scoliosis results: comparison between the groups. There were no real differences in scoliosis results between the two forms of orthosis, apart from sagittal profile outcomes, which were better for the Sforzesco brace. Click here for file

Additional file 3 Clinical results: comparison between the groups. The clinical results almost never reached the point of statistical significance. I: Improved; W: worsened; U: unchanged. Click here for file

Additional file 4 Results according to bone age. We did not find differences between results in the young patients (R2: Risser 2 or less) versus the old ones (R3: Risser 3 ormore). Click here for file

## References

[B1] Aulisa L, Di Benedetto A, Vinciguerra A (1981). [Biomechanical analysis of the spinal brace system in idiopathic scoliosis]. Arch Putti Chir Organi Mov.

[B2] Stagnara P (1976). Les deformations du rachis.

[B3] Stagnara P (1985). Les deformations du rachis.

[B4] Mammano S, Scapinelli R (1992). Plaster casts for the correction of idiopathic scoliosis. Acta Orthop Belg.

[B5] Dickson RA, Leatherman KD (1978). Cotrel traction, exercises, casting in the treatment of idiopathic scoliosis. A pilot study and prospective randomized controlled clinical trial. Acta Orthop Scand.

[B6] Aulisa L, Lupparelli S, Pola E, Aulisa AG, Mastantuoni G, Pitta L (2002). Biomechanics of the conservative treatment in idiopathic scoliotic curves in surgical "grey-area". Stud Health Technol Inform.

[B7] Sibilla P, Negrini S, Rainero G (2002). Trent'anni di scoliosi. Lezione "non" magistrale. Rachide & Riabilitazione 2002.

[B8] Sibilla P, Negrini S, Sibilla P (2001). Il trattamento conservativo attivo della scoliosi idiopatica in Italia. Le deformità vertebrali: stato dell'arte.

[B9] Negrini S, Aulisa L, Ferraro C, Fraschini P, Masiero S, Simonazzi P, Tedeschi C, Venturin A (2005). Italian guidelines on rehabilitation treatment of adolescents with scoliosis or other spinal deformities. Eura Medicophys.

[B10] Mehta MH (2005). Growth as a corrective force in the early treatment of progressive infantile scoliosis. J Bone Joint Surg Br.

[B11] Karger C (1998). Scoliose idiopathique. Encycl Méd Chir – AKOS Encyclopédie Pratique de Médecine.

[B12] Margonato V, Fronte F, Rainero G, Merati G, Veicsteinas A (2005). Effects of short term cast wearing on respiratory and cardiac responses to submaximal and maximal exercise in adolescents with idiopathic scoliosis. Eura Medicophys.

[B13] Romano M, Carabalona R, Petrilli S, Sibilla P, Negrini S (2006). Forces exerted during exercises by patients with adolescent idiopathic scoliosis wearing fiberglass braces. Scoliosis.

[B14] Dickson RA (1985). Conservative treatment for idiopathic scoliosis. J Bone Joint Surg Br.

[B15] Dolan LA, Donnelly MJ, Spratt KF, Weinstein SL (2007). Professional opinion concerning the effectiveness of bracing relative to observation in adolescent idiopathic scoliosis. J Pediatr Orthop.

[B16] Goldberg CJ, Moore DP, Fogarty EE, Dowling FE (2001). Adolescent idiopathic scoliosis: the effect of brace treatment on the incidence of surgery. Spine.

[B17] Negrini S, Marchini G (2007). Efficacy of the Symmetric, Patient-oriented, Rigid, Three-dimensional, active (SPoRT) concept of bracing for scoliosis: a prospective study of the Sforzesco versus Lyon brace. Eura Medicophys.

[B18] Negrini S, Marchini G, Tomaello L (2006). The Sforzesco brace and SPoRT concept (Symmetric, Patient-oriented, Rigid, Three-dimensional) versus the Lyon brace and 3-point systems for bracing idiopathic scoliosis. Stud Health Technol Inform.

[B19] Zaina F, Negrini S, Monticone M, Paroli C, Aulisa AG (2007). Repeatability of the Aesthetic Index for adolescent scoliosis idiopathic evaluation. 4th International Conference on Conservative Management of Spinal Deformities: 13–16 May 2007.

[B20] Risser JC (1976). Scoliosis treated by cast correction and spine fusion. Clin Orthop Relat Res.

[B21] Negrini S (2007). The Evidence-Based ISICO approach to spinal deformities.

[B22] Negrini S (2007). The Evidence-Based ISICO Approach to Spinal Deformities.

[B23] Negrini S, Zaina F, Romano M, Negrini A, Parzini S (2008). Specific exercises reduce brace prescription in adolescent idiopathic scoliosis: A prospective controlled cohort study with worst-case analysis. J Rehabil Med.

[B24] Nachemson AL, Peterson LE (1995). Effectiveness of treatment with a brace in girls who have adolescent idiopathic scoliosis. A prospective, controlled study based on data from the Brace Study of the Scoliosis Research Society. J Bone Joint Surg Am.

[B25] Stagnara P, Mollon G, De Mauroy J (1990). Reeducation des scolioses.

[B26] Negrini S, Negrini A, Romano M, Verzini N, Parzini S (2006). A controlled prospective study on the efficacy of SEAS.02 exercises in preparation to bracing for idiopathic scoliosis. Stud Health Technol Inform.

[B27] Negrini S, Negrini A, Santambrogio GC, Sibilla P, D'Amico M, Merolli A, Santambrogio GC (1995). Relation Between Static Angles of the Spine and a Dynamic Event Like Posture: Approach to the Problem. Three Dimensional Analysis of Spinal Deformities.

[B28] Beauchamp M, Labelle H, Grimard G, Stanciu C, Poitras B, Dansereau J (1993). Diurnal variation of Cobb angle measurement in adolescent idiopathic scoliosis. Spine.

[B29] Zetterberg C, Hansson T, Lindstrom J, Irstam L, Andersson GB (1983). Postural and time-dependent effects on body height and scoliosis angle in adolescent idiopathic scoliosis. Acta Orthop Scand.

[B30] Dawson EG, Smith RK, McNiece GM (1978). Radiographic evaluation of scoliosis: a reassessment and introduction of the scoliosis Chariot. Clin Orthop Relat Res.

[B31] Morrissy RT, Goldsmith GS, Hall EC, Kehl D, Cowie GH (1990). Measurement of the Cobb angle on radiographs of patients who have scoliosis. Evaluation of intrinsic error. J Bone Joint Surg Am.

[B32] Carman DL, Browne RH, Birch JG (1990). Measurement of scoliosis and kyphosis radiographs. Intraobserver and interobserver variation. J Bone Joint Surg Am.

[B33] Ylikoski M, Tallroth K (1990). Measurement variations in scoliotic angle, vertebral rotation, vertebral body height, and intervertebral disc space height. J Spinal Disord.

[B34] Zmurko MG, Mooney JF, Podeszwa DA, Minster GJ, Mendelow MJ, Guirgues A (2003). Inter- and intraobserver variance of Cobb angle measurements with digital radiographs. J Surg Orthop Adv.

[B35] Bunnell WP (1984). An objective criterion for scoliosis screening. J Bone Joint Surg Am.

[B36] Bunnell WP (1993). Outcome of spinal screening. Spine.

[B37] Bunnell WP (2005). Selective screening for scoliosis. Clin Orthop Relat Res.

[B38] Grosso C, Negrini S, Boniolo A, Negrini AA (2002). The validity of clinical examination in adolescent spinal deformities. Stud Health Technol Inform.

[B39] Ferraro C, Gottardo A (1993). La misurazione del gibbo: studio critico mediante un dispositivo tascabile. Minerva Ortop Traumatol.

[B40] Zaina F, Negrini S, Romano M, Aulisa AG (2007). Repeatability of different methods to collect in everyday clinics the sagittal profile of patients with adolescent idiopathic scoliosis. 4th International Conference on Conservative Management of Spinal Deformities: 13–16 May 2007.

[B41] Negrini A, Negrini S, Romano M, Verzini N, Parzini S, Monticone M, Negrini A (2006). A blind radiographic controlled study on the efficacy of Active Self-Correction according to SEAS.02. 3rd International Conference on Conservative Management of Spinal Deformities: 7–8 April 2006.

[B42] Negrini S, Negrini A, Romano M, Verzini N, Parzini S (2006). A controlled prospective study on the efficacy of SEAS.02 exercises in preventing progression and bracing in mild idiopathic scoliosis. Stud Health Technol Inform.

[B43] Brace Wear Compliance. http://www.srs.org/professionals/bracing_manuals/section3.pdf.

[B44] Landauer F, Wimmer C, Behensky H (2003). Estimating the final outcome of brace treatment for idiopathic thoracic scoliosis at 6-month follow-up. Pediatr Rehabil.

[B45] Nicholson GP, Ferguson-Pell MW, Smith K, Edgar M, Morley T (2002). Quantitative measurement of spinal brace use and compliance in the treatment of adolescent idiopathic scoliosis. Stud Health Technol Inform.

[B46] Nicholson GP, Ferguson-Pell MW, Smith K, Edgar M, Morley T (2003). The objective measurement of spinal orthosis use for the treatment of adolescent idiopathic scoliosis. Spine.

[B47] Rahman T, Bowen JR, Takemitsu M, Scott C (2005). The association between brace compliance and outcome for patients with idiopathic scoliosis. J Pediatr Orthop.

[B48] Takemitsu M, Bowen JR, Rahman T, Glutting JJ, Scott CB (2004). Compliance monitoring of brace treatment for patients with idiopathic scoliosis. Spine.

[B49] Vandal S, Rivard CH, Bradet R (1999). Measuring the compliance behavior of adolescents wearing orthopedic braces. Issues Compr Pediatr Nurs.

[B50] Katz DE, Richards BS, Browne RH, Herring JA (1997). A comparison between the Boston brace and the Charleston bending brace in adolescent idiopathic scoliosis. Spine.

[B51] Howard A, Wright JG, Hedden D (1998). A comparative study of TLSO, Charleston, and Milwaukee braces for idiopathic scoliosis. Spine.

[B52] Weiss HR, Weiss GM (2005). Brace treatment during pubertal growth spurt in girls with idiopathic scoliosis (IS): a prospective trial comparing two different concepts. Pediatr Rehabil.

[B53] Bunnell WP, MacEwen GD, Jayakumar S (1980). The use of plastic jackets in the non-operative treatment of idiopathic scoliosis. Preliminary report. J Bone Joint Surg Am.

[B54] Weiss HR, Negrini S, Rigo M, Kotwicki T, Hawes MC, Grivas TB, Maruyama T, Landauer F (2006). Indications for conservative management of scoliosis (guidelines). Scoliosis.

